# Case Report: Pediatric deep vein thrombosis: when pain in the leg becomes a telltale sign

**DOI:** 10.3389/fmed.2026.1909309

**Published:** 2026-10-08

**Authors:** Georgina G. Balyorugulu, Eunice Barnabas, Nshoma Malole, Haniye Almasi, James Makorere

**Affiliations:** 1 Department of Pediatric and Child Health, Kamanga Medics Hospital, Mwanza, Tanzania; 2 Department of Orthopedic, Kamanga Medics Hospital, Mwanza, Tanzania; 3 Department of Radiology, Sekou Toure Regional Zonal Hospital, Mwanza, Tanzania

**Keywords:** deep vein thrombosis, pediatric, trauma, venous thromboembolism, venous thrombosis

## Abstract

**Introduction:**

Deep vein thrombosis is rare in the pediatric population. It carries severe complications such as pulmonary embolism with high mortality. Since it shares presentation with other causes of acute limb pain, diagnosis might be delayed. Early detection of leg pain with immobility, fever, and swelling should raise an alarm for investigating deep vein thrombosis and prompt immediate management. We report a case of a child with chronic thrombocytosis and anemia diagnosed with deep vein thrombosis after sustaining an injury.

**Case presentation:**

Here we report a case of a two-year-old boy presenting with right leg pain for two days with fever after sustaining a fall while playing. x-ray of the leg ruled out fracture, with further investigation showing a raised D-dimer, and a Doppler ultrasound confirming deep vein thrombosis of the right common femoral vein. Standard-of-care anticoagulants were initiated and later discharged on daily oral anticoagulants for six weeks, with scheduled biweekly follow-up clinics and hematologist review.

**Conclusion:**

Deep vein thrombosis, though rare in the pediatric population, requires a high index of suspicion in those presenting with limb pain and risk factors such as trauma. Delay in recognition can lead to catastrophic complications such as pulmonary embolism and death.

## Introduction

Deep vein thrombosis (DVT) is rarely seen in pediatric patients. The prevalence has been estimated to range from 0.07/10000 in general to 3.9–5.1/10000 in admissions (1, 2). DVT mostly affects neonates and adolescents in the pediatric population (3). The incidence of VTE events is much lower in children than in adults, due to reduced thrombin generation, increased alpha-2 microglobulin, which inhibits thrombin and an antithrombotic state of the endothelial wall (4). There are several factors associated with DVT in children, namely, cancer particularly hematological, visceral, and soft tissue such as leukemia and Wilm's tumor, congenital heart disease, trauma, presence of a central venous catheter more so the femoral vein route, decreased intravascular status, birth control pills, obesity, sickle cell anemia, liver failure, immobilization, and infection (5–7).

**Table 1:** Complete blood count on the day of admission.

Examination	Flag	Result	Result unit	Reference range
WBC	H	20.61	10*9/L	3.5–9.5
RBC		4.69	10*12/L	3.8–5.8
PLT	H	1211	10*9/L	150–450
NEUT#		13.91	10*9/L	2.00–6.90
NEUT%		67.5	%	45.0–75.0
MONO#		2.08	10*9/L	0.00–0.90
MONO%	H	10.1	%	2.0–8.0
LYMPH#		4.62	10*9/L	0.60–3.40
LYMPH%		22.4	%	20.0–40.0
IG#		0.29	10*9/L	0.0–0.12
IG%		1.4	%	0.0–1.6
RDW-SD		41.9	fL	41.0–58.0
RDW-CV	H	20.5	%	11.0–16.0
PDW	L	10.9	fL	13.0–31.0
MPV	L	9.1	fL	10.0–16.0
P-LCR	L	15.8	%	24.5–65.5
P-LCC		191	10*9/L	
PCT		1.1	%	0.11–0.37
MCV	L	53.1	fL	80.0–100.0
MCH	L	17.9	pg	27.0–32.0
MCHC		33.7	g/dL	32.0–36.0
HCT	L	24.9	%	37.0–47.0
HGB	L	8.4	g/dL	12.0–16.0

Early detection, management, and prevention offer protection and prevent devastating complications, such as pulmonary embolism, cerebral vascular accidents, and post-thrombotic syndrome. Diagnosis focuses on a high index of suspicion in a patient with risk factors presenting with limb pain. In children, challenges in diagnosis include, scoring systems such as Wells score are used in adults to screen for DVT but have performed poorly in the pediatric population (8) and D-dimer is non-specific and works best for suspecting DVT but not for ruling it out. D-dimer levels can be affected by trauma, viral infections, and inflammatory processes, and tend to fluctuate with age; however, no validated age-adjustment models exist for pediatric cohorts, so in pediatric cases where DVT is suspected, a Doppler ultrasound should be performed whether D-dimer is suggestive or not.

Management includes anticoagulants such as unfractionated heparin (UFH), lower-molecular-weight heparin (LMWH), and vitamin K antagonists (9). Direct oral anticoagulants (DOACs) have recently been used in children with good outcomes (9, 10). Close monitoring and response are crucial in preventing complications and iatrogenic bleeding and preventing long-term morbidity. Here we describe a case of DVT presenting in a toddler with chronic thrombocytosis and anemia after an acute traumatic injury.

## Case presentation

A two-year-old boy presented with right lower limb pain for 2 days after he sustained an injury while playing, although the mother did not witness the injury. The pain was initially accompanied by moderate swelling, with weight-bearing limited on the affected leg. The swelling slightly improved, but the thigh pain persisted and was accompanied by an inability to walk or move the right leg. He also presented with fever for 2 days, generalized body weakness, lower abdominal pain, poor appetite, dry cough, and a runny nose a few days before the fall. On examination, he was alert and irritable, with mild paleness of the conjunctiva noted and stable vitals. He weighed 12 kg with normal weight-for-height percentile. Local examination of the right leg revealed intact skin with no bruising or laceration; tenderness on flexion of the right hip joint and on the anterior medial aspect of the thigh with moderate swelling compared to the left thigh; normal range of movement of the hip and knee joints; and palpable distal pulses. The respiratory, cardiovascular, and abdominal examinations were normal. An x-ray of the pelvis and right femur was ordered to rule out fracture or sprain, which was unremarkable. Malaria rapid diagnostic test was negative and a complete blood count (CBC) revealed leukocytosis 20.61 × 10*9/L, neutrophil count 13.91 × 10*9/L with a neutrophil percentage of 67.5%, thrombocytosis 1211 × 10*9/L, anemia of hemoglobin level of 8.4 g/dL with a low MCV 53.1 fL, low MCH 17.9 pg, low hematocrit 24.9% with an RBC 4.68 × 10*12/L (Table 1). Hb electrophoresis showed Hb AA. Initial diagnosis was fracture of the right femur (which was ruled out by imaging), septic arthritis, septicemia, common cold, and iron deficiency anemia with a reactive thrombocytosis. He was admitted and was kept empirically on a broad-spectrum antibiotic, ceftriaxone 1 g intravenous (IV) once a day, and paracetamol 200 mg IV three times a day for pain, with nasal drops, and cough syrup for the flu. An orthopedic consultation was requested, which ruled out musculoskeletal causes for the pain. The patient continued to have leg pain on the second day post-admission. Further investigations showed serum ALT 20.1 U/L, AST 24.1 U/L, serum creatinine 44 mmol/L, BUN 2.1 mmol/L, prothrombin time 13.5 s, and INR 1.25. Activated partial thromboplastin time was not done. DVT was suspected; a D-dimer was ordered, and the results were >10 mg/L (Reference range 0–0.5 mg/L), raising suspicion for DVT. A Doppler ultrasound of the right leg was performed, which showed non-compressibility of the common femoral vein with intraluminal echogenic thrombus. Color Doppler showed flow ranging from absent to markedly reduced within the affected segments. Spectral Doppler demonstrated loss of normal phasicity and absence of augmentation with distal compression. Posterior tibial and peroneal veins showed reduced flow, consistent with distal venous involvement. The superficial venous system was unremarkable. Impression: Deep vein thrombosis involving the right common femoral vein. He was started on LMWH (enoxaparin) 12 mg at a dose of 1 mg/kg subcutaneously twice daily for 5 days, and oral iron supplementation (Ferrous fumarate syrup) at a dose of 9 mL orally twice a day for three months. DOACs (rivaroxaban) were initiated on the sixth day after completing LMWH. Rivaroxaban suspension was given at a dose of 5 mg orally twice a day; the medication was given as a body weight-adjusted formulation for 6 weeks and was scheduled for a 2-week follow-up clinic. Control CBC after 2 days showed the leukocyte count significantly reduced to 8.64 × 10*9/L but still had thrombocytosis of 1010 × 10*9/L. From the fifth day onward, there was a significant improvement: the pain had resolved, and he was able to walk on his own. He was discharged after eight days of inpatient management. Measurement of anti-Xa levels, which is used to monitor anticoagulant therapy, was not feasible at our facility due to a lack of reagents and equipment. Despite this, we ensured safety during treatment by clinically monitoring the patient and checking for signs of bleeding, namely bruising, hematuria, blood in stool, paleness, and close monitoring of the DVT-affected limb by assessing swelling, pain, limb use, distal pulses and motion. Upon reviewing his medical records, it showed a chronic history of thrombocytosis and anemia; therefore, in addition to the ongoing care, he was also referred to a hematologist for further evaluation. In addition, the parent did not report any familial history of known thrombotic events or similar presentation. Two weeks post discharge follow-up visit showed resolution of thrombocytosis and improved anemia with normal leukocyte count of 9.48 × 10*9/L, a normal platelet count of 364 × 10*9/L and Hb of 9.8 g/dL, and resolution of the DVT on clinical examination, with mother reporting he has resumed his normal activities and playful with no leg pain. Doppler ultrasound to confirm resolution and hematological review are still pending. Counseling was done to emphasis mother ensure the hematogist consultation as recommended.

**Table:** Timeline figure of major events.

Date	Clinical event/action	Category
**DAY 0**	Patient presented with an acute onset right leg pain after an injury while playing	**Symptom onset**
**Day 1**	The pain persisted and mother brought in the child for medical care to the pediatric outpatient clinic	**Diagnosis and evaluation**
	Clinical evaluation- Pallor	
	Local examination of the right leg- normal skin; tenderness on flexion of the right hip joint and on the anterior medial aspect of the thigh with moderate swelling compared to the left thigh; normal range of movement of the hip and knee joints; and palpable distal pulses.	
	Laboratory results: FBP- Leucocytosis 20.61 × 10*9/L with thrombocytosis 1211× 10*9/L, Hb 8.4 g/dL microcytic hypochromic.Hb electrophoresis- Hb AA	
	Right leg x-ray- Unremarkable	
	Orthopedic review- no musculoskeletal issue identified as cause of pain	
	Intervention: Ceftriaxone, paracetamol, IV fluids were initiated,	
**Day 3**	Persistent leg pain with refusal to bear weight and irritability	**Diagnosis and evaluation**
	D dimer - > 10 mg/L (Reference range 0–0.5 mg/L)	
	Doppler USS- Deep vein thrombosis involving the right common femoral vein.	
**Day 4**	Anticoagulants-LMWH (enoxaparin) 12 mg at a dose of 1 mg/kg, subcutaneously twice daily for 5 days and ferrous fumarate syrup −9mls bd for 3 months were initiated	**Intervention**
**Day 9**	Clinically significant improvement with patient ambulating without pain and reduced swelling	**Intervention**
	DOAC-Rivaroxaban suspension was given at a dose of 5 mg per oral twice a day; the medication was given as a body weight adjusted formulation for 6 weeks. Clinical monitoring continued	
**Day 10**	Patient doing well no bleeding diathesis with marked improvement. Discharged with Rivaroxaban. Follow-up every 2 weeks	**Outcome intervention**
**Day 24**	Child is cheerful and active and mother reports no complications with normal activity. Clinically not pale, with normal right leg examination findings	**Follow-up**
	Lab findings- Improvement with resolved thrombocytosis and anemia; CBC showed a normal leukocyte count of 9.48 × 10*9/L, a normal platelet count of 364 × 10*9/L and Hb of 9.8 g/dL.	
	Contorl Doppler USS and hematologist review pending	

## Discussion

DVT mostly affects adults and is rare in children, with a slightly higher male predominance seen in young children (11). In the index case, the patient is a male with a history of chronic thrombocytosis and anemia. Thrombocytosis can be essential, with a higher risk of thrombotic events or reactive, usually secondary to acute or chronic infections, tissue damage from surgery or trauma, iron deficiency anemia, inflammatory disorders, and loss or dysfunction of the spleen. Thus, in this case, trauma, inflammatory process, and iron deficiency anemia might be the culprit in thrombocytosis detected. The fact that the child had chronic thrombocytosis with anemia might also favor iron deficiency as a cause with current acute presentation exacerbating the presentation. Furthermore, patient's platelet count dropped as iron therapy was initiated and evaluated after a two-week follow-up visit. A rapid reduction in platelet count has also been reported in a study (12). In animal models, iron deficiency anemia has shown to induce a hypercoagulable state with thrombocytosis (13). In addition, iron deficiency has been shown to affect vascular blood flow with increased turbulence and reduce red cell deformability, which increases viscosity and activates the coagulation cascade (14). A multi-institutional retrospective study showed that iron deficiency increased risk of VTE by 75% (15). However, thrombotic events with thrombocytosis are rarely seen in pediatric cases. In rare cases of essential thrombocytosis in children, thrombosis occurs in 4%, whereas in adults it occurs in 30% (16).

Thrombocytosis not only poses a risk for clotting events but can also lead to bleeding. A high platelet count, when dysfunctional, has been attributed to interfering with the clotting cascade by breaking down clotting proteins such as von Willebrand factor in cases of acquired von Willebrand syndrome, causing bleeding as a result of essential clonal thrombocytosis (17). In our case, the risk of bleeding was taken into consideration, especially taking into account the use of anticoagulant medication, and challenges in confirming underlying cause of thrombocytosis. Reactive thrombocytosis rarely causes bleeding and was more supported by the clinical presentation, preliminary laboratory results, and clinical response. Nonetheless, close clinical monitoring was implemented for bleeding diathesis.

Sepsis is a risk factor for DVT (5), and infection can result in reactive thrombocytosis. Blood culture and sensitivity for our index case showed no bacterial growth, and CBC showed leukocytosis without neutrophilia, which points to a reactive response to injury and inflammation rather than infection. Also since the child had cough and flu in clinical presentation, viral infection can also be considered. Nonetheless, he was empirically kept on a broad-spectrum antibiotic, anticoagulants, and other supportive management, and improved. Thus, it is important to rule out an infectious cause and treat accordingly.

DVT mostly affects distal veins, the femoral vein, common femoral, popliteal, and iliac veins (18). In our patient, DVT was noted in the right common femoral vein, resulting in pain and tenderness on the anterior medial aspect of the right thigh. Clinical diagnosis in the pediatric population can be missed or delayed due to the lack of awareness of the disease in this age group. Local examination and clinical history guide the diagnosis towards DVT. Scoring systems such as the Wells score are used to screen adults and assess the need for further evaluation. Unfortunately, these screening systems have performed poorly in pediatrics (19). Once clinical history and local examination findings suggest of DVT justifies imaging studies for confirmation (20, 21).

VTE can present as DVT or PE, with some cases having both. In a retrospective study done in South Africa, PE was detected in 28% of eighteen children diagnosed with DVT who presented with unilateral leg pain (22). PE has a mortality rate of 26% (23, 24), and its silent, ominous presentation makes it imperative to rule it out in patients presenting with DVT. Clinical symptoms of PE, such as chest pain, shortness of breath, and hemoptysis, may be absent in 15% of pediatric cases (25). D-dimer levels are used as a screening test for PE; when elevated, a computed tomography pulmonary angiography (CTPA) can be performed to confirm the diagnosis. For our index case, clinical signs or symptoms of PE, such as dyspnea and chest pain, were absent, and the patient had stable vital signs, including oxygen saturation and heart rate. CTPA was not performed because the test was unavailable. Nonetheless, the patient was closely monitored. Upon discharge, he was kept on strict follow-up clinics, and counseling was provided to the mother on signs of PE to return immediately for urgent care.

Management of DVT is mainly with anticoagulants such as LMWH or UFH. The former is much preferred since it offers superior bioavailability, a more predictable pharmacokinetic profile, and a more palatable route of administration (26–28). DOACs have recently gained traction in the management of DVT in the pediatric population (29). The American Society of Hematology and the International Society on Thrombosis and Haemostasis (ASH/ISTH) suggest a much shorter anticoagulant therapeutic duration of 6 weeks for pediatric provoked VTE. The recommendation excludes those presenting with PE, persistent occlusive thrombus, recurrent VTE, cancer-associated thrombosis, major thrombophilia or persistent antiphospholipid antibodies, and ongoing VTE risk factors (30, 31). DOACs have also been shown to be beneficial for thrombus resolution, and the ASH/ISTH recommends their use in pediatric patients (32, 33). Our patient was kept on LMWH for 5 days and switched to DOACs until discharge. DOACs were maintained as outpatient management for 6 weeks, with close monitoring. The challenge we faced in managing anticoagulants is that, at our facility, we were not able to monitor anti-factor Xa levels to ensure they were maintained within the therapeutic range of 0.5–1 IU/mL for the twice-daily LMWH dose, which is checked 4 to 6 h after the subcutaneous injection (34, 35). In the pediatric population, the drug has rapid clearance and may require a higher dose to reach the target therapeutic range; therefore, monitoring is crucial (35, 36). In resource-limited settings where anti-Xa assays are unavailable, meticulous serial clinical examination for bleeding diathesis can serve as a crude primary surrogate for treatment safety.

In the management of DVT, it is important to consider the risk of recurrence to prevent future VTE events. Recurrence of VTE has been reported in 6% to 21%, especially in unprovoked VTE. The index case excluded familial thrombotic events. The case was most likely a provoked DVT with clear risk factors, therefore eliminating but not ruling out the need for thrombophilia genetic screening. It is important to meticulously assess multigenerational family history as a natural diagnostic filter for selecting children for thrombophilia testing. In our case, follow-up, a hematologist review, and monitoring can help guide clinical decisions regarding care and prevention of recurrence.

## Limitations

In this case diagnostic tests such as serum iron, serum ferritin, total iron binding capacity, transferritin for iron deficiency anemia were not available to confirm diagnosis, fibrinogen and CTPA were not done for screening for PE, control Doppler ultrasound was not done due to social economical reasons, not having a hematologist at our centre delayed consultation and review and therefore exhausting causes and risk factors for DVT including management. Despite these challenges important steps where taken to ensure proper safe treatment and follow-up for the child.

## Conclusion

DVT is rare in children but carries dire consequences, such as PE and post-thrombotic syndrome. Trauma has been mentioned as a risk factor for DVT; thus, consider DVT in children presenting with unilateral leg pain, especially after a traumatic injury. A proper diagnostic approach, including history and clinical examination, should be performed, and if DVT is suspected, a Doppler ultrasound should be done. Once confirmed, management with anticoagulants is essential to achieve resolution, prevent complications, and reduce recurrence. Identification of the underlying cause or risk is vital in deciding the treatment approach and whether future prophylactic anticoagulants are required to prevent recurrence. This case highlights the clinical presentation, diagnosis, and management of trauma-provoked DVT, with hiccups in diagnosis and management due to limited resources. To ensure proper care, a multidisciplinary approach with paediatricians, an orthopedic specialist, a radiologist, and a hematologist was used to achieve a good outcome.

## Data Availability

The original contributions presented in the study are included in the article/Supplementary Material, further inquiries can be directed to the corresponding author.
